# Supplementary material for: Geographical distribution, disease association and diversity of Klebsiella pneumoniae K/L and O antigens in India: roadmap for vaccine development
Source: Microb Genom. 2024 Jul 22;10(7):001271. doi: 10.1099/mgen.0.001271 (PMC11316559; doi:10.1099/mgen.0.001271)
Supplement: Uncited Supplementary Material 1. [file mgen-10-01271-s001.pdf]

Supplementary Material 1: Results from the de-replicated assemblies

1. **Overview**

To evaluate the biases in our dataset caused by clonal expansion, we conducted the same analysis after removing identical or highly similar genome sequences.

2. **De-replication Process**

Using the Assembly Dereplicator tool (https://github.com/rrwick/Assembly-Dereplicator), we de-replicated our initial dataset of 1072 genomes. This tool identifies and removes redundant assemblies based on sequence similarity, ensuring that unique genome sequences are retained for further analysis. For de-replication, we used a MASH-distance of 0.001.

3. **Results**

The initial dataset contained 1072 genomes. After the de-replication process, 312 non-duplicated genomes remained. The metadata for the dereplicated genomes is provided in Supplementary table 12.

4. **Diversity of KL and O antigens**

We conducted a diversity analysis on the de-replicated dataset to assess the distribution of serotypes, sequence types (STs), and virulence factors. The dereplication process, which decreased the dataset from 1072 to 312 genomes, had a minimal impact on the overall diversity of KL types, retaining 75 out of the original 78 types. The additional three KL types identified in the whole collection were unknown types, suggesting they were not truly new types, but were probably the result of incomplete assemblies. The order of the top KL types slightly changed post-dereplication, while KL64 remained the predominant type, KL2 and KL51 switched order.

The total number of unique O-locus types after dereplication was 12, compared to 13 in the original dataset. Comparable to the KL types mentioned above, the additional O type identified in the whole collection was an unknown type, suggesting it was not truly a new type, but rather the result of an incomplete assembly. The two most common O-locus types, O1/O2v1 and O1/O2v2 remained predominant after de-replication. The relative proportions of less common O-locus types like O3b, OL101, O3/O3a, and O4 slightly changed.

While the diversity of KL and O types remained similar, the proportion of KL types differed, suggesting that some KL types (namely KL51) were predominant due to clonal expansion. In contrast, others are more widespread in the *K. pneumoniae* population (for example KL64).

Table 1. Distribution of K Loci in de-replicated *Klebsiella pneumoniae* genomes.

Sl. No	K_locus	Count (n)	Sl. No	K_locus	Count (n)
1	KL64	48	39	KL23	2
2	KL2	31	40	KL28	2
3	KL51	23	41	KL3	2
4	KL10	16	42	KL38	2
5	KL81	14	43	KL48	2
6	KL62	12	44	KL63	2
7	KL24	9	45	KL74	2
8	KL1	8	46	KL103	1
9	KL112	8	47	KL104	1
10	KL17	8	48	KL106	1
11	KL102	7	49	KL116	1
12	KL15	6	50	KL122	1
13	KL105	5	51	KL127	1
14	KL108	5	52	KL128	1
15	KL21	5	53	KL13	1
16	KL110	4	54	KL136	1
17	KL16	4	55	KL140	1
18	KL25	4	56	KL142	1
19	KL36	4	57	KL148	1
20	KL52	4	58	KL150	1
21	KL114	3	59	KL169	1
22	KL139	3	60	KL173	1
23	KL20	3	61	KL177	1
24	KL27	3	62	KL186	1
25	KL30	3	63	KL19	1
26	KL54	3	64	KL22	1
27	KL57	3	65	KL34	1
28	KL109	2	66	KL35	1
29	KL115	2	67	KL39	1
30	KL117	2	68	KL47	1

31	KL125	2	69	KL55	1
32	KL14	2	70	KL58	1
33	KL146	2	71	KL67	1
34	KL153	2	72	unknown (KL104)	1
35	KL154	2	73	unknown (KL28)	1
36	KL155	2	74	unknown (KL31)	1
37	KL158	2	75	unknown (KL68)	1
38	KL183	2		**Total**	**312**

Abbreviations: Sl., serial number.

Table 2. Distribution of O Loci in de-replicated *Klebsiella pneumoniae* genomes.

**O_locus**	**Count (n)**
O1/O2v1	142
O1/O2v2	81
O3b	27
OL101	24
O3/O3a	15
O4	12
O5	5
OL103	2
O1/O2v3	1
O12	1
OL104	1
unknown (O1/O2v1)	1
Grand Total	312

In the original dataset of 1072 *K. pneumoniae* isolates, a vaccine formulation incorporating 5 O types would cover 90% of isolates across various specimen types and disease conditions. Similarly, a vaccine targeting 25 KL types would potentially cover up to 90% of the Indian *K. pneumoniae* population, including 85% of the carbapenem-resistant *K. pneumoniae* (CRKP) population. However, in the de-replicated data, we observed a reduction in coverage, with a vaccine incorporating 25 KL types only covering 75% of the overall population while still covering 85% of the CRKP isolates. Despite this reduction in KL type coverage, incorporating

5 O types would still cover 90% of the population, consistent with the original dataset (Figure S6).

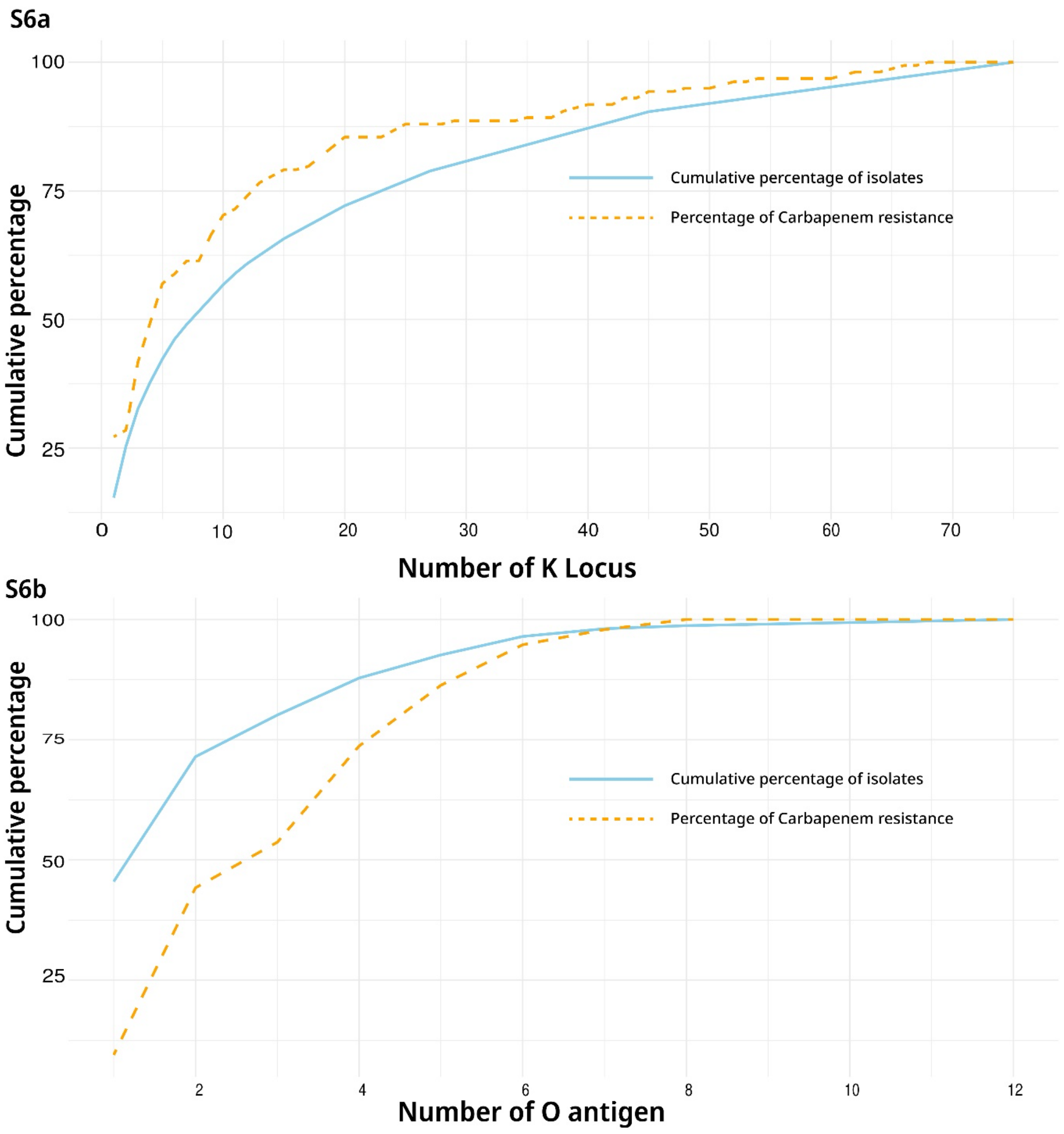

Supplementary Figure 6. The cumulative coverage of the KL and the O types represents the percentage of de-replicated genomes covered. The KL and O types are sorted with the highest to lowest frequency. The orange dotted line shows cumulative carbapenemase-positive de-replicated genomes.

5. **Serotypes and STs associated with them**

After de-replication, we identified 113 STs, which is nearly as diverse as the 114 STs observed in the original dataset. However, the predominant STs changed notably. ST147 emerged as the most predominant ST, while ST231, which was the most predominant ST in the original dataset, became the third most frequently found (Table 3).

ST147 exhibited heterogeneity in the de-replicated genomes by hosting more than three KL types, but the number of ST147 genomes with KL51 was considerably lower (0.6% (2/312) in the de-replicated dataset vs. 1.6% (20/1072) in the original dataset). ST231, which predominantly carried KL51, was clonal, and de-replication removed this clonally spreading ST (20% (213/1072) in the original compared to 4% (14/312) in the de-replicated genomes), thereby altering the distribution of STs in the dataset (Table 4).

Other major STs in the original dataset, such as ST395-KL64, ST14-KL2, and ST2096-KL64, were also less prevalent in the de-replicated genomes, suggesting these clones were primarily spread by clonal expansion in India.

Table 3. Major ST counts from the de-replicated dataset. ST_other represents minor STs with a frequency of less than 5.

ST	Grand Total
ST147	41
ST15	18
ST231	18
ST16	15
ST11	14
ST14	12
ST395	12
ST23	7
ST307	7
ST101	6
ST48	6
ST_other	156
**Total**	**312**

Table 4. K Locus count and distribution of STs among each KL type. The KL types and STs with a frequency lesser than 6 are grouped as ST_other and KL_other respectively.

K-Locus	ST	Count
KL64	ST_other (n=13), ST14 (n=1), ST147 (n=21), ST231 (n=4), ST395 (n=9)	48
KL2	ST_other (n=10), ST101 (n=2), ST11 (n=3), ST14 (n=11), ST15 (n=5)	31
KL51	ST_other (n=1), ST147 (n=2), ST16 (n=6), ST231 (n=14)	23
KL10	ST_other (n=3), ST147 (n=13)	16
KL81	ST_other (n=4), ST11 (n=1), ST147 (n=1), ST16 (n=8)	14
KL62	ST_other (n=6), ST48 (n=6)	12
KL24	ST_other (n=1), ST11 (n=4), ST15 (n=4)	9
KL1	ST_other (n=1), ST23 (n=7)	8
KL112	ST15 (n=8)	8
KL17	ST_other (n=5), ST101 (n=3)	8
KL102	ST_other (n=1), ST307 (n=6)	7
KL15	ST_other (n=4), ST11 (n=2)	6
KL_other	ST_other (n=107), ST101 (n=1), ST11 (n=4), ST147 (n=4), ST15 (n=1), ST16 (n=1), ST307 (n=1), ST395 (n=3)	122

6. Regional distribution of KL and O antigens in de-replicated genomes

After de-replication, the diversity indices for the different regions showed some variation, but the overall trends were maintained. Specifically, the diversity indices of KL types remained higher in the Southern and Central regions even after de-replication.

Table 5. Simpson Diversity index of KL types among different regions.

Region	De-replicated data Simpson Diversity Index
Central	0.916
Eastern	0.915
North-Eastern	0.875
Northern	0.845
Southern	0.962
Western	0.903

7. Virulence factors associated with capsular (KL) and O antigen serotypes

Among the 48 KL64 isolates, 73% (35/48) of them carried yersiniabactin with a virulence score of 1, only 14% of them carried yersiniabactin and aerobactin with virulence score of 4, and 10% (5/48) of them had a virulence score of 0. In the de-replicated genomes, KL51 isolates exhibited lower virulence scores. Specifically, 65% carried only yersiniabactin with a virulence score of 1, and only 21% (5/23) carried both hypervirulence markers yersiniabactin and aerobactin.

The proportion of KL51 isolates carrying aerobactin decreased substantially from 70% in the original dataset to 21% in the de-replicated genomes. For KL2 and KL1, the virulence score distribution and the virulence factors remained the same as seen in the original dataset. Despite the reduction in genome numbers, the same 13 other KL types carrying the hypervirulence marker aerobactin were observed as in the original dataset, indicating that the reduction of duplicate genomes did not affect the presence of hypervirulent clones, but only altered their proportions.

Although the Kruskal-Wallis rank sum test revealed a significant association between virulence scores and certain KL types (Kruskal-Wallis chi-squared = 143.25, p-value <0.05) in the de-replicated population, no significant association was found between the presence of any virulence factors with the KL types in the de-replicated dataset. This contrasts with the original dataset, where a significant association between yersiniabactin and aerobactin with certain KL types was observed. This indicates that de-replication altered the observed relationships between specific factors and KL types, suggesting that duplicate genomes may have influenced the initial associations.

8. Resistance profiles associated with capsular (KL) and O antigen

The de-replicated data showed a slightly higher proportion of multidrug resistance at 79% (246/312). However, the proportion of isolates exhibiting carbapenem resistance dropped to 56% (176/312) in the de-replicated dataset. Moreover, only 50% (158/312) of the de-replicated

genomes had a resistance score of 2. This suggests that while the overall MDR rate remained consistent, the prevalence of carbapenem resistance and resistance score 2 was notably lower in the de-replicated genome set. We observed a similar distribution of resistance scores among the KL types as compared to the original 1072 genomes. Despite the reduction in genomes, the genes driving the carbapenem resistance and their association with KL types remained largely consistent with the original dataset. This consistency suggests that the de-replication process did not significantly alter the observed patterns of resistance gene carriage across different KL types. In the de-replicated dataset, we found that incorporating 4 O types would cover 75% of the carbapenem-resistant population, while approximately 20 KL types would cover 90% of the carbapenem-resistant genomes (Figure S6).

Supplementary Figures

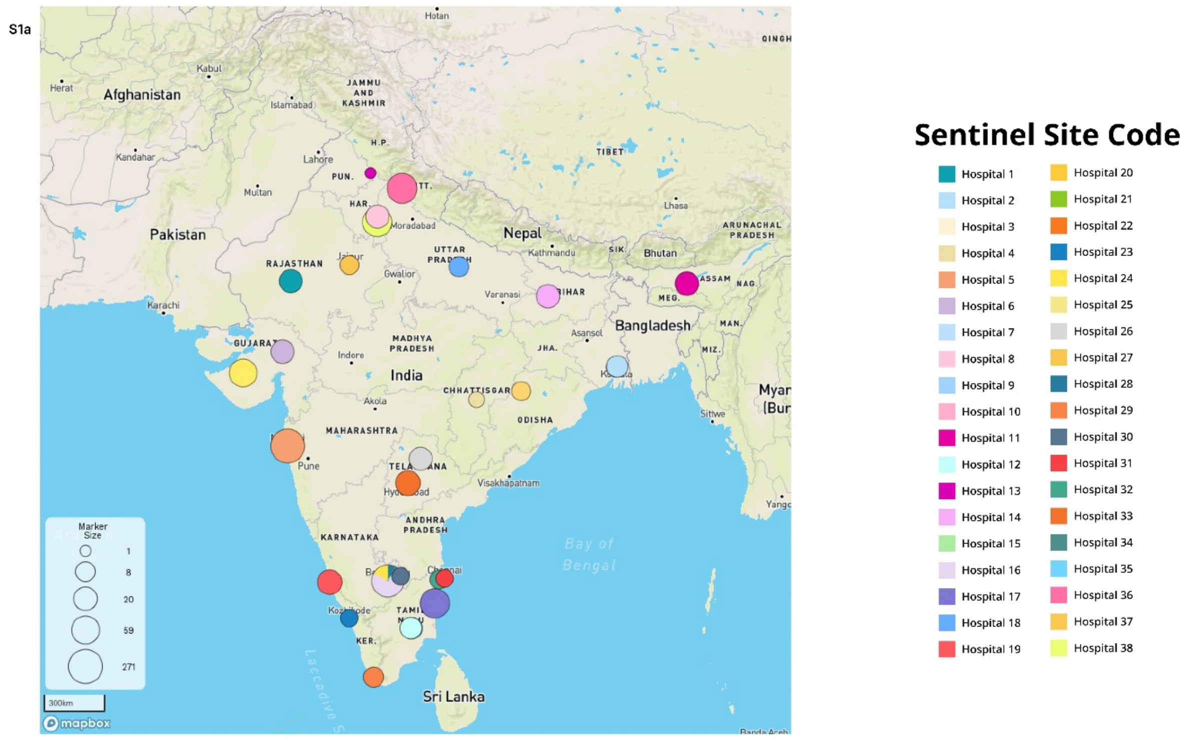

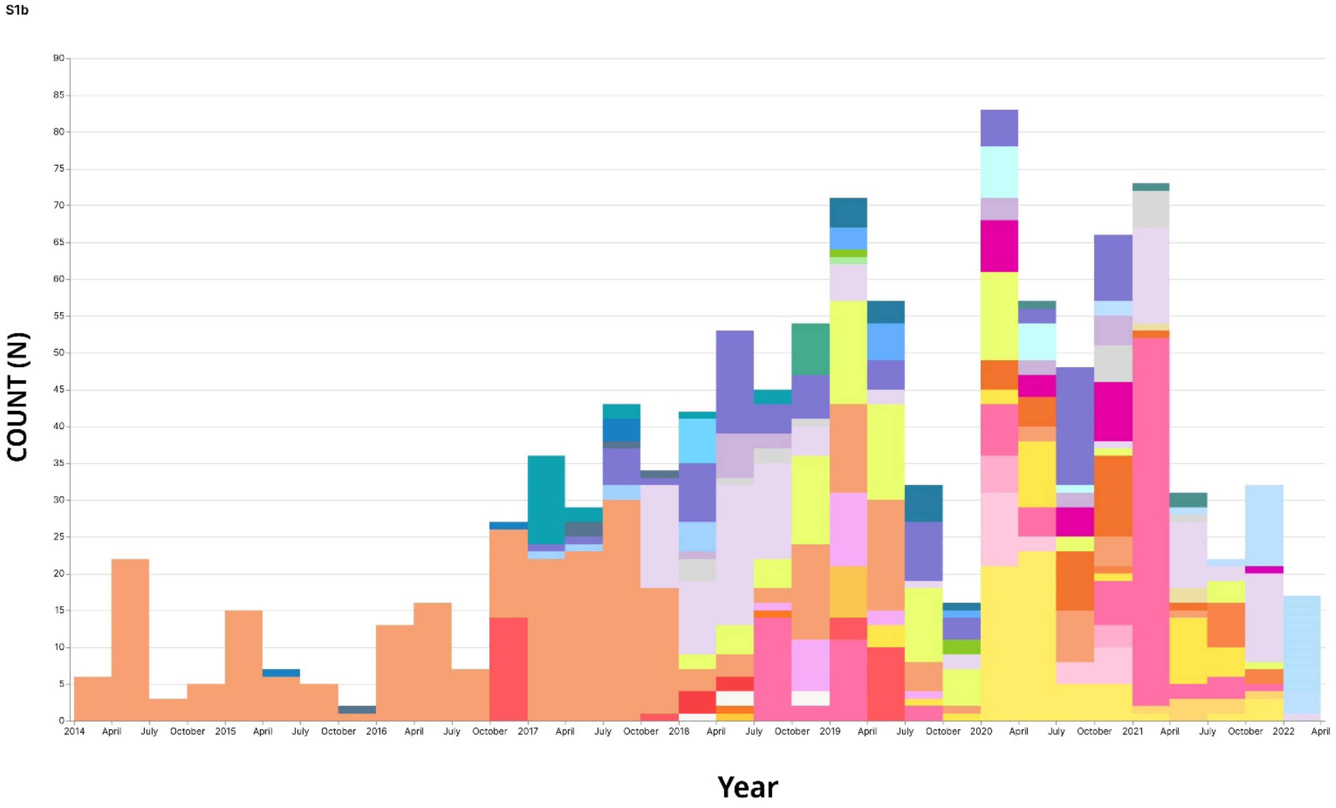

Supplementary Figure 1A: Geographical distribution of the 1072 *K. pneumoniae* isolates collected across 38 sentinel sites in India.

Supplementary Figure 1B: Timeline of the samples collected from 2013 to 2022 for this study.

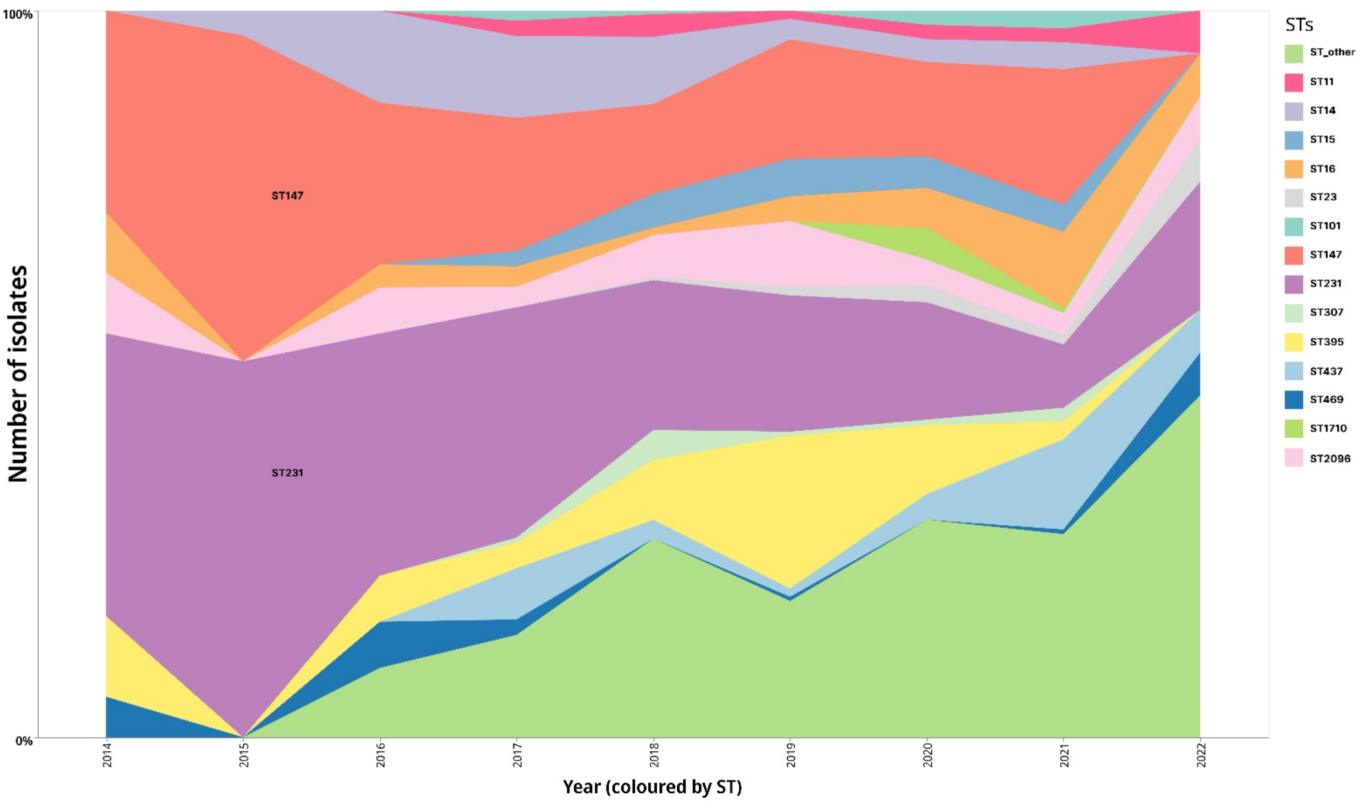

Supplementary Figure 2: Year-wise distribution of STs identified across the study period of 2014-2022. STs having <14 entries are grouped as ST_other.

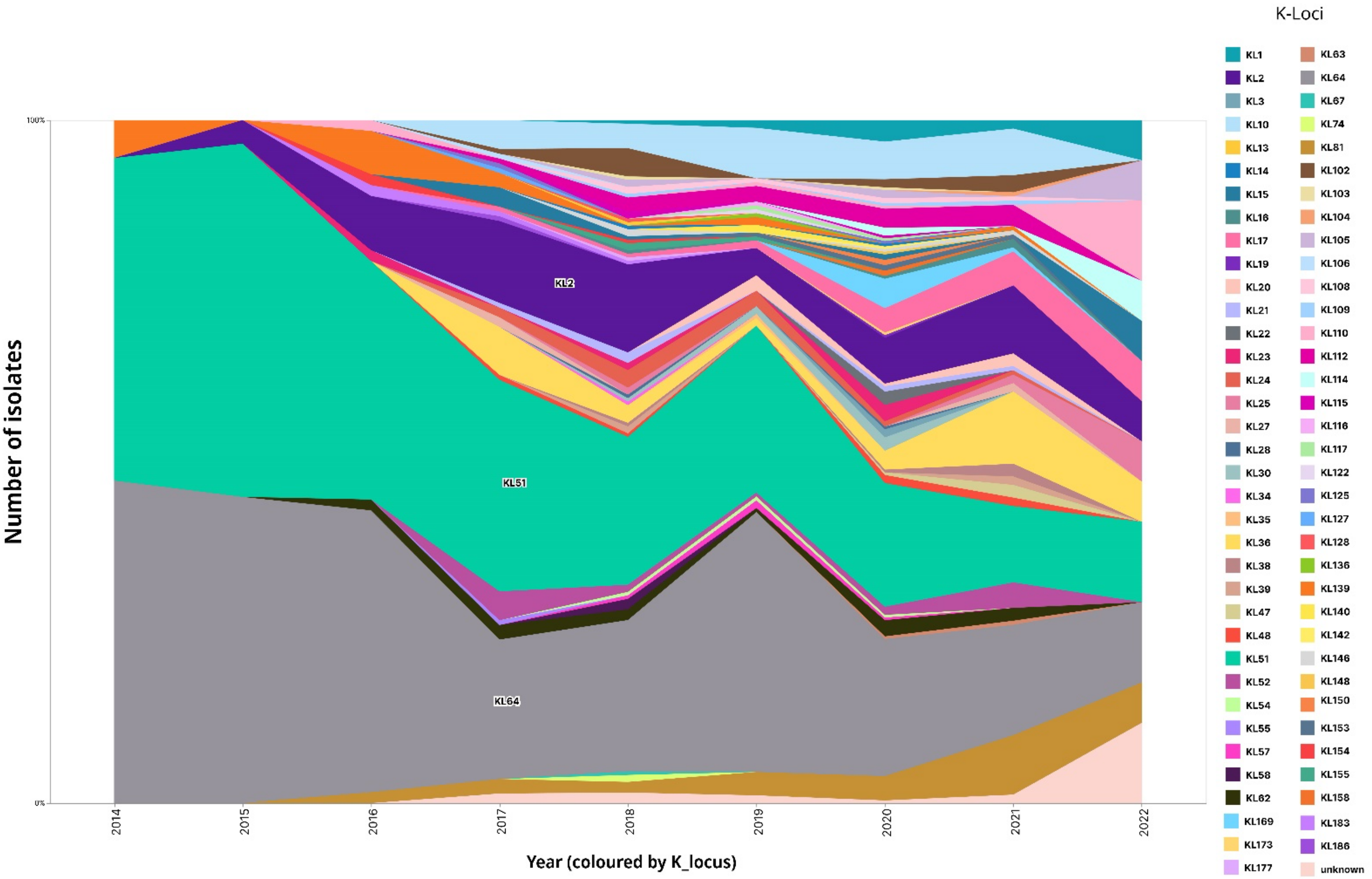

Supplementary Figure 3: Year-wise distribution of K Locus types identified across the study period of 2014-2022.

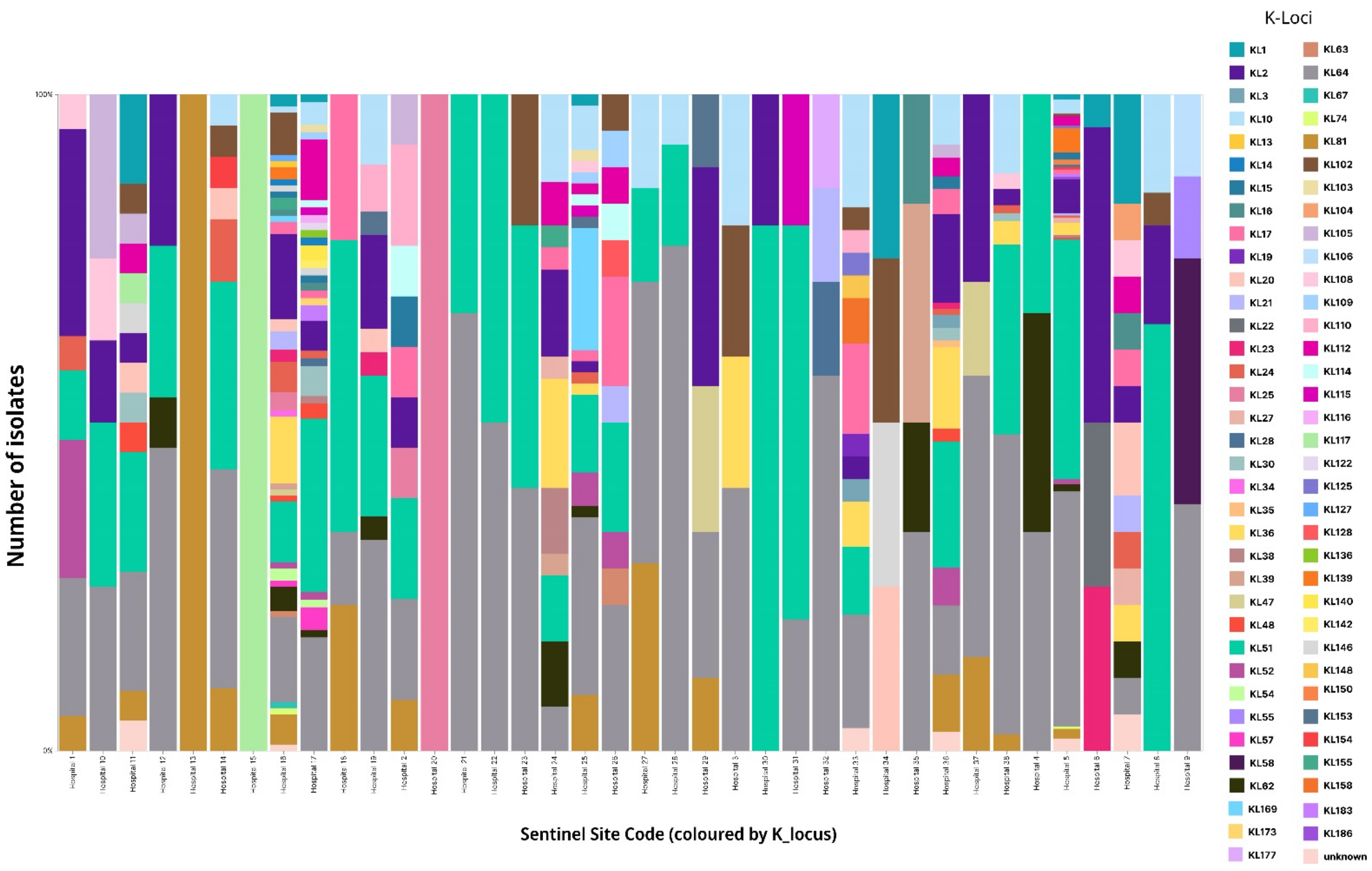

Supplementary Figure 4: Distribution of K Locus types identified across each sentinel site. KL types are stacked to 100% within each site.

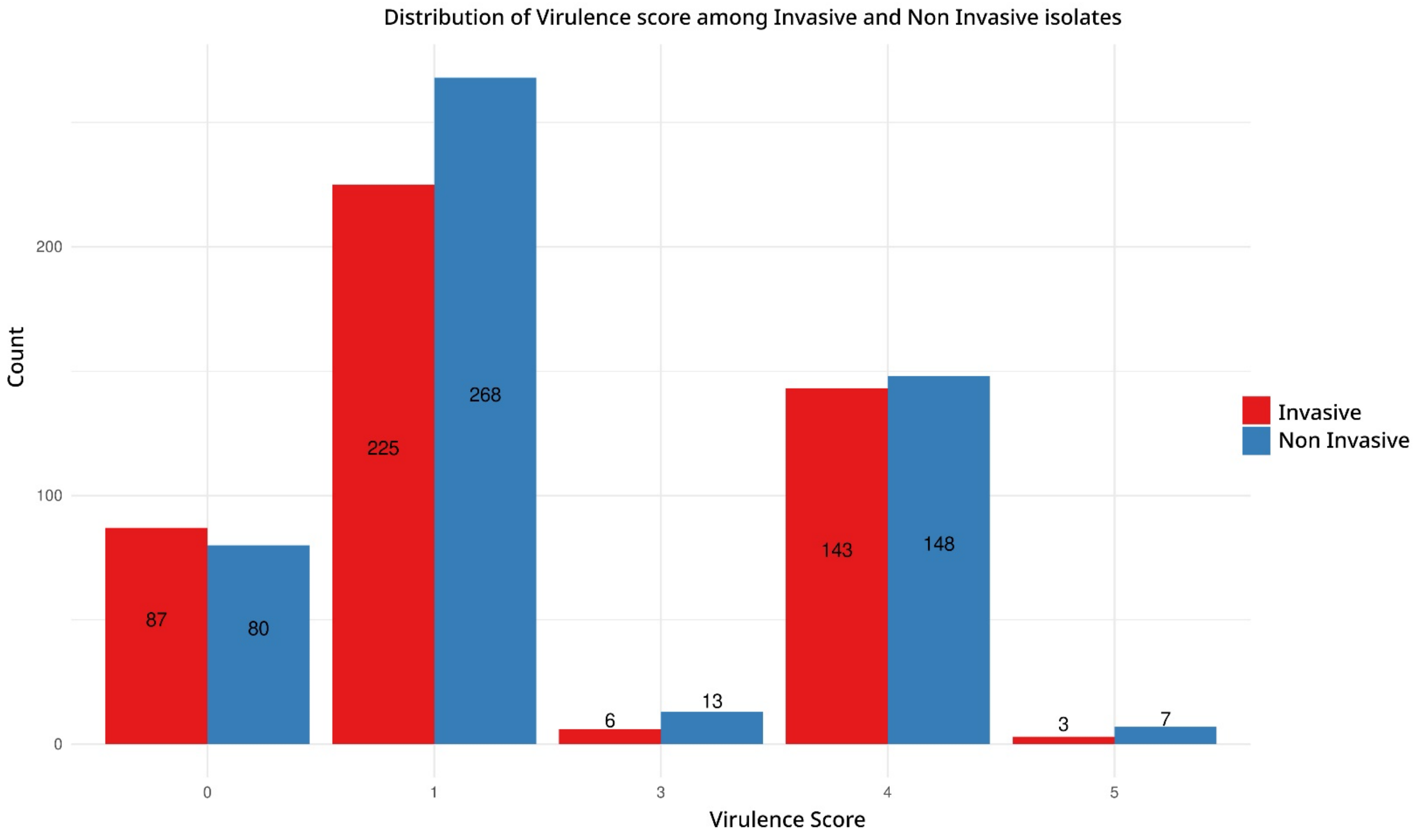

Supplementary Figure 5: Distribution of Virulence score among the invasive and non-invasive isolates.
